# Weight loss is a sufficient and economical single outcome measure of murine dextran sulfate sodium colitis

**DOI:** 10.1096/fba.2019-00035

**Published:** 2019-07-01

**Authors:** Savini Lanka Britto, Mahesh Krishna, Richard Kellermayer

**Affiliations:** ^1^ Section of Pediatric Gastroenterology Texas Children's Hospital Baylor College of Medicine Houston Texas; ^2^ USDA/ARS Children's Nutrition Research Center Houston Texas

**Keywords:** animal model, mice, mouse

## Abstract

Inflammatory bowel diseases (IBD: Crohn's disease and ulcerative colitis) are becoming common around the world without a cure. Animal models of colitis have become instrumental in IBD research. The dextran sulfate sodium (DSS) induced murine colitis model is likely the most utilized due to its simplicity and reproducibility with over 4000 publications on PubMed, where weight loss is the most commonly used and reliable positive correlate. We predicted at current state of art, that the DSS colitis model can be optimized by using weight loss as a single cost‐saving outcome measure. Twenty recent and consecutive publications using the DSS model in PubMed were selected for review. Guarded cost estimations for additional outcome measures of colitis beyond weight loss were performed. In all manuscripts (100%), weight loss corroborated the conclusions. Average excess cost for examining additional measures of colitis was approximately $6700 per publication. Two studies (10.5%) were estimated to have spent over $20,000 in excess. Additional measures of colitis either supported the final conclusions found with weight loss, or lead to indeterminate results. Potential annual savings from following our guidance were calculated to be over $60,000 for and IBD lab. We conclude that weight loss is a sufficient, objective, and economical outcome measure of DSS‐induced colitis in mice.

**Abbreviations**
APC: antigen‐presenting cell
CD: Crohn's disease
DAI: disease activity index
DSS: dextran sulfate sodium
ELISA: enzyme‐linked immunosorbent assay
IBD: Inflammatory bowel disease
MPO: myeloperoxidase
SCFA: short‐chain fatty acid
TUNEL: terminal deoxynucleotidyl transferase dUTP nick end labeling
UC: ulcerative colitis

## 1 INTRODUCTION

Inflammatory bowel diseases (IBD), Crohn's disease (CD) and ulcerative colitis (UC) are chronic, relapsing‐remitting inflammatory disorders of the gastrointestinal tract. The exact etiology of IBD is still unknown, but appears multifactorial and influenced by genetics, host immune dysfunction, mucosal barrier defects, and the gut microbiome.^1^, ^2^ Many animal models, particularly mouse models, of IBD have been developed to better understand the pathophysiology of IBD and to pre‐clinically explore therapies to be used in humans.^3^, ^4^, ^5^, ^6^ Perhaps the most commonly used mouse model of colitis is the administration of dextran sulfate sodium (DSS) to the animals. DSS is a water soluble, negatively charged sulfated polysaccharide with a highly variable molecular weight. The exact mechanism of DSS‐induced colitis is not clearly defined, but its major effect is disruption of the intestinal barrier integrity leading to intestinal inflammation.^7^, ^8^, ^9^ The severity of the resulting colitis can be altered by many factors including, but not limited to the strain or gender of the animal or the molecular weight of DSS selected.^10^, ^11^ While of significant value in basic scientific studies, the DSS‐induced colitis model is acknowledged to be imperfect, and to mostly capture/model the intestinal injury and recovery associated with inflammatory responses of IBD.^9^, ^12^

DSS colitis most closely resembles human UC,^13^ however, findings from this model are still translated back to IBD in general, which includes CD. Although this difference remains, many therapeutic interventions effective in human CD have been reported to be efficacious against DSS‐induced colitis.^3^ Furthermore, the DSS‐induced colitis model remains very popular in IBD research particularly due to its simplicity, reliability, and efficiency (relatively short timeframe to obtain results). It can be used as a model for acute, chronic, and relapsing intestinal inflammation simply by adjusting the DSS concentration and frequency of administration. Mice susceptible to DSS typically have a noticeable weight loss, generally about 5%‐10%, within 3‐4 days of DSS administration.^14^ Weight loss greater than 20% of initial weight is a physiological indicator of animal stress and possibly imminent demise.^15^ Graphing percent change in body weight over time is the standard way to depict disease progression in this colitis model.^10^ Weight loss is almost always used to identify which mice are exhibiting a more severe colitis. In fact our impression, after more than a decade of work with this model, is that weight loss has been a consistent and highly reliable outcome measure of DSS colitis in over 1000 publications. However, much too often, additional redundant testing is performed to further support the weight loss‐related conclusion of colitis severity. Supplementary investigations of other measures of colitis are generally costly and time consuming. These include, but are not limited to, histological analysis with scoring by a pathologist, cytokine expression at the mRNA and protein levels, and other indirect measures of colitis activity such as tissue myeloperoxidase (MPO) analysis.

IBD carries significant economic burden worldwide due to its rising prevalence, high morbidity, and costs associated with research and treatment options, particularly biologic agents.^16^, ^17^, ^18^ It has become increasingly important to practice cost‐effective medicine due to rising healthcare costs,^19^, ^20^ which in part is linked to basic research spending.^21^ Numerous studies have analyzed and suggested solutions to reduce the economic burden of IBD targeting healthcare costs,^22^, ^23^, ^24^, ^25^ but we propose that scrutinizing research costs can be of indirect benefit as well. Our aim was to highlight the unnecessary excess cost associated with research utilizing a DSS‐induced colitis murine model.

## 2 MATERIALS AND METHODS

A literature search on “dextran sulfate sodium colitis” was performed using PubMed in October 2018. Over 4000 articles resulted from this search. Twenty 20 of the most recently published articles at that time were selected (Supplementary Table S1). Articles excluded were those utilizing cancer models of DSS, those not assessing the severity of DSS‐induced colitis, or those we did not have access to.

Measures utilized to assess colitis severity were recorded for each article. Testing performed for mechanistic purposes (ie, examining molecular mechanisms involved in DSS colitis) or for other objectives beyond colitis severity assessment were not included. Only measures of colitis severity beyond weight loss were included in the cost estimation. For details of cost estimation, see Supplementary Methods. Additionally, see Supplementary Table S1 for detailed cost analysis breakdown.

## 3 RESULTS

**Figure 1:**
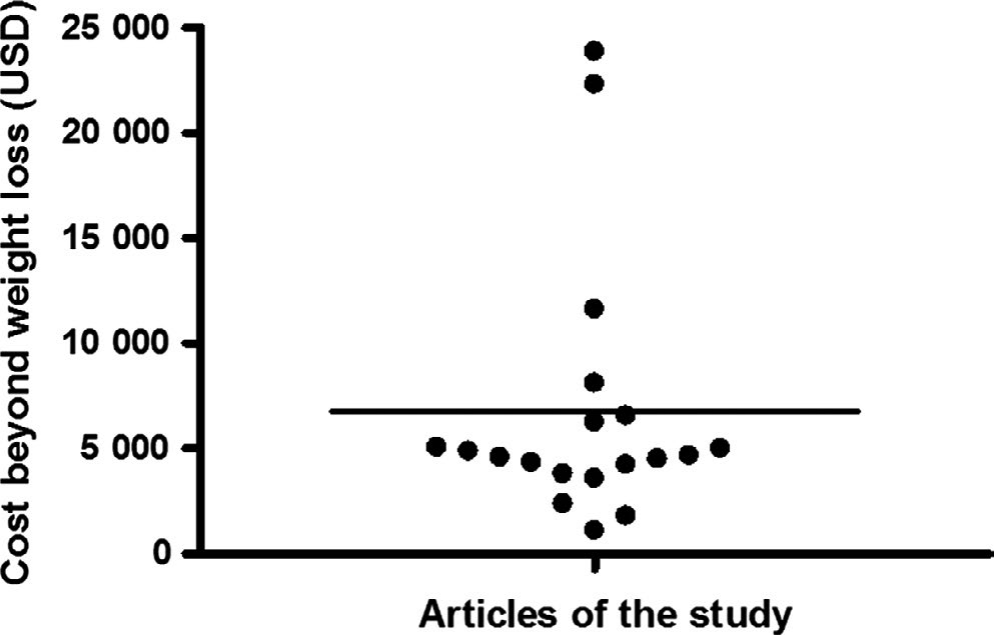
Cost estimation for outcome measures of DSS colitis beyond weight loss in consecutive articles published prior to October 2018 (n = 19). Average excess cost was approximately $6700 ($1092.31‐23 874.62)

Among the 20 consecutive articles included, one paper that documented monitoring weights in their methods was unclear about how weight change was incorporated into assessing disease activity.^26^ The remaining 95% of papers had weight loss as a clear and important measure of DSS colitis incorporated into their findings. Figure 1 depicts our highly guarded cost estimate analysis of the 19 articles. The average excess cost beyond weight loss assessment among these papers was approximately $6,700. Two^2^ studies were estimated to have spent over $20 000.^27^, ^28^ The average impact factor (in 2017) of the journals where the manuscripts of this study were published was approximately 3.94. The cost of the added colitis severity measures beyond weight loss did not correlate with the journal of publication's impact factor (r = −0.12; *P* = 0.627).

Among the 20 studies included, 19 performed histological analysis. If not otherwise documented, studies were assumed to have used one pathologist for assigning histological scores (n = 17). Two studies indicated using two^29^ and three^30^ blinded pathologists. In all cases, histological score was consistent with weight loss (100% consistency for histologic scoring). Disease activity index (DAI) scores were utilized in many of the studies (n = 16) for which weight loss was always a component of the score and DAI always correlated with weight loss alone (100% consistency for DAI). Studies that included cytokine analyses (n = 15) varied in many ways, especially how cytokines were assessed. Samples were from serum or colonic tissue and they were tested via enzyme‐linked immunosorbent assay (ELISA), mRNA expression, antigen presenting cell (APC) and/or western blot. The conclusions from these studies also varied. Most found that their cytokine analysis was consistent with weight loss (n = 10), while one^1^ had indeterminate results, one^1^ did not correlate and the remaining overlapped with consistent and indeterminate (n = 3) findings (86.7% consistency for cytokine analysis). Those with any form of indeterminate results were all from studies that assessed four or more cytokines. Results of MPO analysis (n = 5) were always consistent with weight loss (100% consistency with MPO).

Additional studies not commonly performed included flow cytometry (n = 3), immunohistochemistry (n = 3), terminal deoxynucleotidyl transferase dUTP nick end labeling (TUNEL) assay for apoptosis (n = 2), short‐chain fatty acid analysis SCFA (n = 1), and fecal calprotectin analysis (n = 1). These tests added considerable cost and the findings were all consistent with weight loss (100% consistency with cell‐based analyses). Additional tests performed that did not add extra costs included colon length (n = 14) and weight measurements (n = 2) along with spleen weight (n = 4). These were all consistent with weight loss, except one study that found no difference in colon weight, one study that found no difference in colon length and one study that found no difference in spleen weight (85% consistency with objective organ‐based assessments).

Therefore, other measures of colitis in the DSS‐induced murine model of colitis were found to either support the initial findings (average consistency of additional colitis measures with weight loss = 94%) based on weight loss alone or resulted in indeterminate results.

## 4 DISCUSSION

In this work, we examined if weight loss may be a sufficient single outcome measure of DSS colitis in most IBD research. This question was raised based on our impression after more than a decade of work with this model.^31^, ^32^ We found that weight loss is the most consistently used outcome of the model (95% of studies clearly used it, while 5% likely without clear description of the disease activity index used^26^). Several studies (n = 16) included DAI to evaluate the clinical progression of colitis. This is not a standardized tool. Among publications, the components of DAI measurements varied. For instance, some used weight loss, stool consistency, and visual blood in the stool.^27^, ^33^ Other variations included weight loss, stool consistency and visual rectal bleeding^34^ or weight loss, stool consistency, general appearance, and visual rectal bleeding.^35^ Therefore, the only objectively measured component within these DAIs was weight loss. Based on discussions with colleagues in the field, DAI measurements are not blinded, hence the remaining factors assessed beyond weight loss are subjective and prone to bias. Another argument would be to utilize macroscopic evaluation (eg, colon shortening), a variable we reported not to carry an extra cost. However, this measurement is subjective as well and technician dependent. If performed incorrectly, such as by stretching the colon or measuring from the top of the cecum rather than the ileocecal junction, then measurements can be inaccurate. Therefore, we emphasize that the results of weight loss alone always corroborated the final conclusion of the manuscripts studied, which was our subjective conclusion from the prior literature as well. Namely, none of the authors of this publication have been able to identify a single publication on DSS colitis in over 1‐10 years of work in the field, where weight loss was inconsistent with the final conclusions on colitis severity.

Nevertheless, all of the consecutive 20 manuscripts examined from 2018 within this work had at least one additional outcome measure investigated. These additional investigations led to a minimum of $6700 average added cost (based on United States of America cost estimates) per publication. In the meantime, the additional measures did not modify the final conclusions of the studies, and in 95% of the cases those corroborated the weight loss findings. Importantly, these extra investigations only addressed colitis severity and did not add any novel conclusions about the biology or pathogenesis of IBD. The extra cost spent did not improve the impact of the papers either, based on the journal citation indices of the publications.

An average lab with modest research activity may complete about 10 DSS experiments per year. Based on our cost estimations, if additional outcome measures beyond weight loss are excluded then the potential annual savings are approximately $67,000 per research lab. To help put this into perspective, private foundation grants in IBD generally offer $100 000 per year for a new project in the USA, so more than half of the grant money could go toward unnecessary excess spending. Hence, the annual savings associated with utilizing weight loss as the sole outcome measure of colitis severity in the DSS colitis model are significant.

Animal models in IBD research are used to address basic science (pathogenesis) questions, evaluate pre‐clinical efficacy, study pharmacokinetics, test safety, and identify biomarkers.^12^ In the case of the 20 consecutive publications studied, 10 (50%) were in the preclinical efficiency category, 9 (45%) in basic science, and 1 (5%) in biomarker identification. We argue, that in the case of the DSS colitis model (and likely in other animal models of IBD as well) weight loss is a sufficient, practical (real‐time, reliable, objective) and cost‐effective outcome measure of colitis severity for the basic science and the pre‐clinical treatment efficiency studies. As emphasized in the methods section, this conclusion does not pertain to testing performed for mechanistic purposes (ie, examining molecular mechanisms involved in DSS colitis) or for other objectives beyond colitis severity assessment.

We trust that our message from this review work will help to optimize future IBD research utilizing DSS and other murine models toward efficiency and cost‐effectiveness.

## CONFLICT OF INTEREST

All authors declare they have no conflicts of interest to disclose.

## AUTHOR CONTRIBUTIONS

SB wrote first draft and performed extensive literature search. MK performed extensive literature search. RK conceptual design, literature search, funding, critical revisions, and final formatting. All authors approved the final version of the manuscript for publication.
