# Supplementary material for: Weight loss is a sufficient and economical single outcome measure of murine dextran sulfate sodium colitis
Source: FASEB Bioadv. 2019 Jul 1;1(8):493–7. doi: 10.1096/fba.2019-00035 (PMC6996316; doi:10.1096/fba.2019-00035)
Supplement: Supplementary file 1 [file FBA2-1-493-s001.docx]

**Supplementary Table**

| Article | Excess Cost Estimation Beyond Weight Loss | Mice (n) | 2017 Journal Citation reports Impact factor |
| --- | --- | --- | --- |
| 1[^1^](#_ENREF_1) | **$4,580.85** | 8 groups, n=6 | 9.504 |
| 2[^2^](#_ENREF_2) | **$4,867.46** | 4 groups (n=5) | 3.457 |
| 3[^3^](#_ENREF_3) | **$1,772.31** | data combined from 3 separate experiments: total mice 40 for acute (over 4 different time points)- day 0, 5, 8 &11; 12 mice for chronic colitis | 5.595 |
| 4[^4^](#_ENREF_4) | **$2,373.08** | 4 groups (N=12) | 4.539 |
| 5[^5^](#_ENREF_5) | **$23,874.62** | 4 groups (n=6 per DSS group, N= 6 per time point), 7 time points total. mice euthanized at each | 3.3 |
| 6[^6^](#_ENREF_6) | **$8,087.62** | 3 groups (12 mice per group) | 2.749 |
| 7[^7^](#_ENREF_7) | **$4,485.35** | 4 groups  (n=10mice per group)- number varies N=5-10 throughout paper | 1.782 |
| 8[^8^](#_ENREF_8) | **$5,061.85** | 4 groups (n = 10) | 4.235 |
| 9[^9^](#_ENREF_9) | **$4,988.87** | 5 groups (n=8) | 0.786 |
| 10[^10^](#_ENREF_10) | **$4,666.62** | 8 groups (n=8) | 3.466 |
| 11[^11^](#_ENREF_11) | **$3,782.85** | 2 groups, repeated 5 times but total of 80 mice) | 4.347 |
| 12[^12^](#_ENREF_12) | **$6,243.08** | 12 groups (total number of mice 130) | 2.766 |
| 13[^13^](#_ENREF_13) | **$11,616.72** | 4 groups (5 time points for euthanizing day 0, 2,4,10, 13), each with 6 mice | 2.109 |
| 14[^14^](#_ENREF_14) | **$4,223.62** | 3 groups (11, 15 and 10 mice) | 9.5 |
| 15[^15^](#_ENREF_15) | **$3,572.08** | 40 mice total- 4 groups (2 experimental and 2 controls) | 4.122 |
| 16[^16^](#_ENREF_16) | **$6,543.85** | 4 groups mice - 1 control, 3 given DSS; 10 mice per group | 2.766 |
| 17[^17^](#_ENREF_17) | **$4,317.08** | 6 groups- 8 mice per group | 2.766 |
| 18[^18^](#_ENREF_18) | **$0.00** | 5 groups, each experiment performed 3 times (n=10) | 3.12 |
| 19[^19^](#_ENREF_19) | **$22,295.08** | Several experiments performed at least 3 times- total mice at first, 48649 to determine which mutation resulted in increased colitis susceptibility. Then only a subset were subjected to histology and cytokines | 4.398 |
| 20[^20^](#_ENREF_20) | **$1,092.31** | 1 part DSS study. n =21, but histology only for n = 18 | 2.63 |

**Supplementary Methods**

Those studies that provided clear information about materials used such as manufacturer name, catalog number of a specific kit (i.e., Enzyme-linked immunosorbent assay (ELISA), MPO assay, etc) were charged the price of 1 kit as shown online as a means to obtain a more accurate cost estimation. If the specific kit was not documented, then the cost of the cheapest kit found online was used. No shipping costs were included. Studies that performed cytokine analysis used samples from either serum or colonic tissue and they were tested via ELISA, mRNA expression, APC and/or Western blot.

Those that included assessment of blood in the stool via fecal occult blood tests (FOBT) were added an extra charge based on $40 per box of 100 FOBT cards and the number of boxes charged was dependent on the total number of mice in the study. This assumes that each mouse had at least one FOBT performed during the study, but does not take into account serial FOBTs. Studies which utilized flow cytometry were charged $50 per antibody used and then $3 per sample based on an affordable flow cytometry technique described in 2005[^21^](#_ENREF_21).

We also incorporated the cost of a lab technician to account for the use of a medical lab technician, cytogenic technologist or histo-technologist, which the studies were presumed to utilize. We chose the lowest salary reported by the American Society for Clinical Pathology in 2015 as the standard to avoid overestimations[^22^](#_ENREF_22). In general, medical/clinical laboratory technicians make the least and the state of Alabama had the lowest reported salary at $17.75/hr or 36,920/year. To account for overhead costs, we approximated the yearly salary for a technician to be $60,000, which translates to approximately $29/hr based on a 40 hour work week. For simplicity we used this salary for all tests that required any type of technician and assumed a full day of work (8 hours total) for each test that was added. Tests included that required a lab technician: cytokine expression via ELISA and/or polymerase chain reaction (PCR), Western Blot, MPO, terminal deoxynucleotidyl transferase dUTP nick end labeling (TUNEL) assay, flow cytometry, fecal calprotectin, and short chain fatty acid analysis (SCFA).

Lastly, most articles included histological analysis. For this, we estimated the cost per tissue sample as $20, which includes processing, staining, and slide materials. We assumed that 1 pathologist per article was employed unless it was openly stated that multiple pathologists were used for histological grading. We used $180,000/year as the approximation for an entry level pathologist, which translates to approximately $87/hr based on a 40-hour work week. If histology was performed, we added the cost of a pathologist for 1 day (8 hours).

**References**

**1.** Gemechu Y, Millrine D, Hashimoto S, et al. Humanized cereblon mice revealed two distinct therapeutic pathways of immunomodulatory drugs. *Proc Natl Acad Sci U S A.* Nov 13 2018;115(46):11802-11807.

**2.** Chi JH, Kim YH, Sohn DH, Seo GS, Lee SH. Ameliorative effect of Alnus japonica ethanol extract on colitis through the inhibition of inflammatory responses and attenuation of intestinal barrier disruption in vivo and in vitro. *Biomed Pharmacother.* Dec 2018;108:1767-1774.

**3.** Reade S, Williams JM, Aggio R, et al. Potential role of fecal volatile organic compounds as biomarkers of chemically induced intestinal inflammation in mice. *FASEB J.* Oct 25 2018:fj201800076RR.

**4.** Nanda SK, Nagamori T, Windheim M, et al. ABIN2 Function Is Required To Suppress DSS-Induced Colitis by a Tpl2-Independent Mechanism. *J Immunol.* Dec 1 2018;201(11):3373-3382.

**5.** Nunes NS, Kim S, Sundby M, et al. Temporal clinical, proteomic, histological and cellular immune responses of dextran sulfate sodium-induced acute colitis. *World J Gastroenterol.* Oct 14 2018;24(38):4341-4355.

**6.** Wasilewska E, Zlotkowska D, Wroblewska B. Yogurt starter cultures of Streptococcus thermophilus and Lactobacillus bulgaricus ameliorate symptoms and modulate the immune response in a mouse model of dextran sulfate sodium-induced colitis. *J Dairy Sci.* Jan 2019;102(1):37-53.

**7.** Xie X, Ni Q, Zhou D, Wan Y. Rab32-related antimicrobial pathway is involved in the progression of dextran sodium sulfate-induced colitis. *FEBS Open Bio.* Oct 2018;8(10):1658-1668.

**8.** Yu T, Wan P, Zhu XD, et al. Inhibition of NADPH oxidase activities ameliorates DSS-induced colitis. *Biochem Pharmacol.* Dec 2018;158:126-133.

**9.** Kim MS, Kim JY. Ginger attenuates inflammation in a mouse model of dextran sulfate sodium-induced colitis. *Food Sci Biotechnol.* Oct 2018;27(5):1493-1501.

**10.** Huang Y, Guo J, Gui S. Orally targeted galactosylated chitosan poly(lactic-co-glycolic acid) nanoparticles loaded with TNF-a siRNA provide a novel strategy for the experimental treatment of ulcerative colitis. *Eur J Pharm Sci.* Dec 1 2018;125:232-243.

**11.** Fan TJ, Tchaptchet SY, Arsene D, et al. Environmental Factors Modify the Severity of Acute DSS Colitis in Caspase-11-Deficient Mice. *Inflamm Bowel Dis.* Oct 12 2018;24(11):2394-2403.

**12.** Mahalhal A, Williams JM, Johnson S, et al. Oral iron exacerbates colitis and influences the intestinal microbiome. *PLoS One.* 2018;13(10):e0202460.

**13.** Kinoshita Y, Arita S, Murazoe H, Kitamura K, Ashizuka S, Inagaki-Ohara K. Subcutaneously administered adrenomedullin exerts a potent therapeutic effect in a murine model of ulcerative colitis. *Hum Cell.* Jan 2019;32(1):12-21.

**14.** Yang Z, Li Q, Wang X, et al. C-type lectin receptor LSECtin-mediated apoptotic cell clearance by macrophages directs intestinal repair in experimental colitis. *Proc Natl Acad Sci U S A.* Oct 23 2018;115(43):11054-11059.

**15.** Liu W, Zhang Y, Qiu B, Fan S, Ding H, Liu Z. Quinoa whole grain diet compromises the changes of gut microbiota and colonic colitis induced by dextran Sulfate sodium in C57BL/6 mice. *Sci Rep.* Oct 8 2018;8(1):14916.

**16.** Sun Y, Zhong S, Yu J, et al. The aqueous extract of Phellinus igniarius (SH) ameliorates dextran sodium sulfate-induced colitis in C57BL/6 mice. *PLoS One.* 2018;13(10):e0205007.

**17.** Sudirman S, Hsu YH, He JL, Kong ZL. Dietary polysaccharide-rich extract from Eucheuma cottonii modulates the inflammatory response and suppresses colonic injury on dextran sulfate sodium-induced colitis in mice. *PLoS One.* 2018;13(10):e0205252.

**18.** Fang R, Wu R, Zuo Q, et al. Sophora flavescens Containing-QYJD Formula Activates Nrf2 Anti-Oxidant Response, Blocks Cellular Transformation and Protects Against DSS-Induced Colitis in Mouse Model. *Am J Chin Med.* Oct 4 2018:1-15.

**19.** McAlpine W, Wang KW, Choi JH, et al. The class I myosin MYO1D binds to lipid and protects against colitis. *Dis Model Mech.* Sep 27 2018;11(9).

**20.** Beaudry K, Langlois MJ, Montagne A, Cagnol S, Carrier JC, Rivard N. Dual-specificity phosphatase 6 deletion protects the colonic epithelium against inflammation and promotes both proliferation and tumorigenesis. *J Cell Physiol.* May 2019;234(5):6731-6745.

**21.** Imade GE, Badung B, Pam S, et al. Comparison of a new, affordable flow cytometric method and the manual magnetic bead technique for CD4 T-lymphocyte counting in a northern Nigerian setting. *Clin Diagn Lab Immunol.* Jan 2005;12(1):224-227.

**22.** Garcia E, Fisher PB. The American Society for Clinical Pathology's 2015 Wage Survey of Medical Laboratories in the United States. *Am J Clin Pathol.* Apr 1 2017;147(4):334-356.
